# Two Chromatin Remodeling Activities Cooperate during Activation of Hormone Responsive Promoters

**DOI:** 10.1371/journal.pgen.1000567

**Published:** 2009-07-17

**Authors:** Guillermo Pablo Vicent, Roser Zaurin, A. Silvina Nacht, Ang Li, Jofre Font-Mateu, Francois Le Dily, Michiel Vermeulen, Matthias Mann, Miguel Beato

**Affiliations:** 1 Centre de Regulació Genòmica (CRG), Universitat Pompeu Fabra, Parc de Recerca Biomèdica (PRBB), Barcelona, Spain; 2 Department of Proteomics and Signal Transduction, Max Planck Institute for Biochemistry, Martinsried, Germany; 3 Department of Physiological Chemistry and Cancer Genomics Centre, University Medical Center Utrecht, Utrecht, The Netherlands; RIKEN Genomic Sciences Center, Japan

## Abstract

Steroid hormones regulate gene expression by interaction of their receptors with hormone responsive elements (HREs) and recruitment of kinases, chromatin remodeling complexes, and coregulators to their target promoters. Here we show that in breast cancer cells the BAF, but not the closely related PBAF complex, is required for progesterone induction of several target genes including MMTV, where it catalyzes localized displacement of histones H2A and H2B and subsequent NF1 binding. PCAF is also needed for induction of progesterone target genes and acetylates histone H3 at K14, an epigenetic mark that interacts with the BAF subunits by anchoring the complex to chromatin. In the absence of PCAF, full loading of target promoters with hormone receptors and BAF is precluded, and induction is compromised. Thus, activation of hormone-responsive promoters requires cooperation of at least two chromatin remodeling activities, BAF and PCAF.

## Author Summary

In order to adapt its gene expression program to the needs of the environment, the cell must access the information stored in the DNA sequence that is tightly packaged into chromatin in the cell nucleus. How the cell manages to do it in a selective maner is still unclear. Here we show that, in breast cancer cells treated with the ovarian hormone progesterone, the hormone receptor recruits to the regulated genes two chromatin remodeling complexes that cooperate in opening the chromatin structure. One of the complexes puts a mark in a chromatin protein that anchors the other complex, enabling full gene activation. The present discovery highlights the importance of the concerted order of events for access to genomic information during activation of gene expression and reveals the intricacies of hormonal gene regulation.

## Introduction

Regulation of eukaryotic gene expression implies mechanisms that permit transcription factors to gain access to chromatin packaged DNA sequences. The basic unit of chromatin, the nucleosome, consists of an octamer formed of two copies of each of the four core histones (H2A, H2B, H3, and H4) around which 147 bp DNA is wrapped in 1.65 left-handed superhelical turns [1]. Modulation of the structure and dynamics of nucleosomes is an important regulatory mechanism in all DNA-based processes and is catalyzed by chromatin remodeling complexes. Such complexes can either modify histone residues or use the energy of ATP hydrolysis to alter the relationship between histones and DNA [2],[3].

The yeast SWI/SNF complex, the first ATP-dependent chromatin remodeling complex to be identified, is a 2-MDa complex of 11 subunits that regulates gene expression by catalyzing octamer transfer, nucleosome sliding, dinucleosome formation, and H2A/H2B displacement [4]–[6]. RSC, (Remodel the Structure of Chromatin) [7], is a closely related yeast chromatin remodeling complex of 15 subunits, that shares two identical and at least four homologous subunits with the ySWI/SNF complex [8]. There are two human SWI/SNF-like complexes both containing ATPase subunits similar to yeast Swi2/Snf2, hBRM (human Brahma) or BRG1 (Brahma-Related Gene 1), as well as a series of other subunits, some of which differ in various cell types [8]. The hSWI/SNF-α complexes, also called BAF for BRG1/hBRM-Associated Factor, contain either hBRM or BRG1 as ATPase and is orthologue to yeast SWI/SNF. The hSWI/SNF-β complex, also called PBAF (Polybromo-associated BAF) contains only hBRG1 and is orthologue to yeast RSC complex [4],[9]. The BAF and PBAF complexes share many subunits but have also subtype specific subunits: BAF250 and hBRM are only found in BAF, whrereas BAF180 and BAF 200 are only found in PBAF [4],[10]. BAF57 has been reported as a common subunit for BAF and PBAF complexes [4],[9].

Histone acetyltransferases (HATs) and histone deacetylases (HDACs) represent other group of chromatin remodeling complexes that regulate the level of acetylation on the N-terminal tails of core histone proteins and other protein substrates [11],[12]. The HATs are divided into five families, including the GCN5-related N-acetyltransferases (GNATs) with GCN5 and PCAF as the best characterized members; the MYST ('MOZ, Ybf2/Sas3, Sas2 and Tip60)-related HATs; p300/CREB-binding protein (CBP) HATs; the general transcription factor HATs including the TFIID subunit TBP-associated factor-1 (TAF1); and the nuclear hormone-related HATs SRC1 and ACTR (SRC3) [13],[14]. Recombinant PCAF, like full-length GCN5, acetylates either free histones or nucleosomes [15], primarily on lysine -14 of histone H3 [16]. The role of PCAF in transcription has been investigated in multiple studies, and its requirement as a HAT and coactivator has been described in several processes including nuclear receptor mediated events [17],[18], but the precise mechanism of action has not yet been elucidated.

The functional relationship between different chromatin remodeling enzymatic activities is of great interest. A remarkable interdependence has been described during transcriptional activation in *S. cerevisiae* between the SWI/SNF complex and the histone acetylation complex SAGA [19]. Bromodomains of the RSC complex have been shown to recognize acetylated H3K14 [20]. HAT activity stabilizes SWI/SNF binding to promoter nucleosomes providing a mechanistic basis for the ordered recruitment of these complexes [21]. In mammalian cells, transcriptional activation by nuclear receptors requires multiple cofactors including CBP/p300, SWI/SNF and Mediator. The ordered recruitment of these cofactors to the promoters depends not only on the direct interactions between nuclear receptors and cofactors but also on cofactor-cofactor interaction and on histone modifications [22].

Gene regulation by steroid hormones is mediated by intracellular receptors, which upon hormone binding can activate signal transduction cascades and interact in the nucleus with other transcription factors and/or with dedicated DNA sequences, called hormone responsive elements (HREs) [23]. When ligand-receptor complexes interact with HREs they alter the transcriptional state of the associated genes via the recruitment of chromatin remodeling complexes and other coregulators to the target promoters [24],[25]. The mouse mammary tumor virus (MMTV) long terminal-repeat region encompasses a promoter including within 140 bp five degenerated hormone responsive elements (HREs) and a binding site for nuclear factor 1 (NF1). In chromatin the MMTV promoter is organized into positioned nucleosomes [26], with a nucleosome located over the five HREs and the NF1 binding site [27]. On this promoter nucleosome, the binding site for NF1 is not accessible and only two of the five HREs, the strong palindromic HRE1 and the weak half palindrome HRE4, can be bound by hormone receptors, while the central HREs, in particular the palindromic HRE2 and the half palindrome HRE3, are not accessible for receptor binding [28]. Following hormone induction *in vivo* all HREs and the binding site for NF1 are occupied simultaneously on the surface of a nucleosome-like structure and a functional synergism is observed between progesterone receptor (PR) and NF1 [27].

Already 5 minutes after addition of hormone to breast cancer cells with an integrated copy of an MMTV-luc reporter, the cytoplasmic signaling cascade Src/Ras/Erk is activated via an interaction of PR with the estrogen receptor, which activates Src [29]. As a consequence of Erk activation, PR is phosphorylated, Msk1 is activated, and a ternary complex PR-Erk-Msk1 is recruited to nucleosome B [30]. Msk1 phosphorylates H3 at serine 10, a modification which is accompanied by acetylation at H3 lysine 14, displacement of HP1γ, and recruitment of RNA polymerase II [30]. Inhibition of Erk or Msk activation blocks H3S10 phosphorylation, H3K14 acetylation, and hormonal induction of MMTV and other progesterone target genes [30].

There have been several reports indicating a role for SWI/SNF, Brg1 and Brm in glucocorticoid regulation of MMTV transcription [31]–[33], but the situation with progesterone is less clear. The MMTV promoter assembled in minichromosomes with positioned nucleosomes [34] is activated by PR in a process involving a NURF-dependent mutual synergism between PR and NF1 [35]. Progesterone treatment of a breast cancer cell line carrying a single integrated copy of a MMTV transgene leads to recruitment of PR, SWI/SNF, and SNF2h-related complexes to the MMTV promoter, accompanied by selective displacement of histones H2A and H2B from nucleosome B [6],[25]. Recently, the acidic N terminus of the Swi3p subunit of yeast SWI/SNF was identified as a novel H2A-H2B-binding domain required for ATP-dependent H2A/H2B dimer displacement [36]. Moreover, a characteristic and sharp DNase I hypersensitive site appears near the symmetry axis of the nucleosome encompassing the HREs [27]. The same hypersensitive site can be induced in the absence of hormone by treatment with low concentrations of histone deacetylases inhibitors, such as sodium butyrate or trichostatin A [37], suggesting that it can be initiated by a moderate increased in histone acetylation. However, the relationship between histone acetylation and ATP-dependent nucleosome remodeling following hormone treatment is not clear. To approach this question we have performed knockdown and ChIP experiments targeting human SWI/SNF complexes and histone acetyl transferases (HATs) in breast cancer cells.

## Results

### BAF is required for progesterone gene activation in T47D-MTVL cells and is recruited to the MMTV promoter upon induction

**Figure 1:**
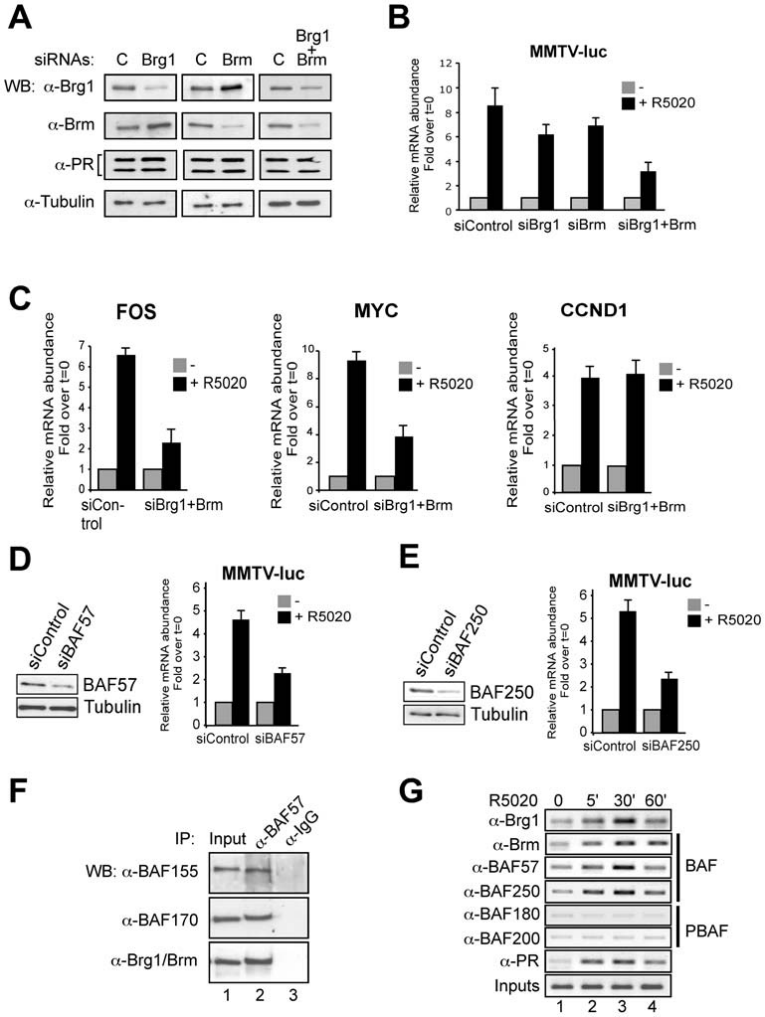
BAF is essential for MMTV promoter activity in T47D-MTVL cells. (A) Cells were transfected either with siRNA against Brg1, Brm , or both siRNAs combined as indicated. After 48 h the medium was replaced by fresh medium without serum. After one day in serum-free conditions, cells were lysed and the levels of Brm, Brg1, PR and tubulin were determined by Western blotting. C, control siRNA. (B) Cells were transfected with Control, Brg1 and Brm siRNAs as described in (A). After one day in serum-free conditions, cells were incubated with 10 nM R5020 for 8 hs and total RNA was prepared, cDNA was generated and used as template for real time PCR with luciferase oligonucleotides. The values represent the mean and standard deviation from 3 experiments performed in duplicate. (C) Cells were transfected with Control, Brg1 and Brm siRNAs as described in (A) and treated with 10 nM R5020. cDNA was generated and used as template for real-time PCR using specific c-Fos, c-Myc, Cyclin D1 and GAPDH primers. The values represent the mean±SD of 3 experiments performed in duplicate. (D) Cells were transfected with Control and BAF57 siRNAs as described in (A) and treated with 10 nM R5020. Left: BAF57 levels were analyzed by Western blotting. Right: RNA was extracted, cDNA was generated and used as template for real time PCR with luciferase oligonucleotides. The values represent the mean±SD of 2 experiments performed in duplicate. (E) Experiments similar to those shown in (D), but cells were transfected with Control and BAF250 siRNAs as described in (A) and (D). The values represent the mean±SD of 2 experiments performed in duplicate. (F) Cells were lysed and immunoprecipitated either with α-BAF57 antibody or with normal rabbit IgG as a negative control (IgG). The immunoprecipitates (IP) were analyzed by western blotting with non-discriminating Brg1/Brm antibody, α-BAF155 or α-BAF170 specific antibodies. (G) Cells were untreated (0) or treated for 5, 30 or 60 min with 10 nM R-5020 and subjected to ChIP assays with α-PR, α-Brg1, α-Brm, α-BAF57, α-BAF250, α-BAF180 and α-BAF200 specific antibodies. The precipitated DNA fragments were subjected to PCR analysis to test for the presence of sequences corresponding to the MMTV nucleosome B. Input material (1%) is shown for comparison. A representative of three independent experiments is shown.

The requirement of Brm and Brg1 for MMTV promoter activation was assessed in T47D-MTVL cells transfected with specific siRNAs. When siRNA against Brg1 was used, the levels of Brm protein increased (Figure 1A, left panels) and viceversa (Figure 1A, middle panels). A significant reduction (80% and 55% for Brm and Brg1, respectively) of both ATPases was only observed when the two siRNAs were combined (Figure 1A, right panels). The levels of the two PR isoforms, PR_B_ and PR_A_, were unaffected by Brg1 or Brm siRNAs (Figure 1A, third row). In cells transfected with control siRNA an eight-fold increase in MMTV-luc transcription was observed 8 hours after hormone treatment (Figure 1B). In cells transfected with siRNAs against either Brg1 or Brm, the induction was reduced by 30% and 20% respectively, whereas transfection of both siRNA simultaneously reduced the induction of the MMTV promoter by 60%, confirming the essential role of hSWI/SNF on MMTV promoter activation (Figure 1B). Progestin induction of MYC and FOS, two other progesterone target genes, was also impaired by the combined siRNAs to a level similar to that observed with MMTV-luc (Figure 1C). However, not all hormone-dependent genes required SWI/SNF, as the combined siRNAs did not inhibit the progestin induction of the cyclin D1 gene (Figure 1C).

To identify the nature of the Brm/Brg1-containing complex in T47D-MTVL cells, we performed western blotting against subunits of BAF and PBAF, the two distinctive hSWI/SNF complexes. Subunits of both complexes exist in T47D-MTVL cells (Figure S1), indicating that our cell line is capable of forming both BAF and PBAF. The specific subunits that distinguish the two complexes are Brm and BAF250 for BAF, and BAF180 and BAF200 for PBAF. Knocking down BAF57 and BAF250 levels by approximately 50% resulted in a similar reduction in the hormonal induction of the MMTV promoter without changes in Brg1 and Brm protein levels (Figure 1D and 1E and data not shown). Co-immunoprecipitation experiments demonstrated that in T47D-MTVL cells BAF57 forms a complex with the core subunits BAF155, BAF170, BAF 180 and the ATPases Brg1 and Brm (Figure 1F, lane 2 and data not shown). We conclude that BAF and PBAF complexes in the breast cancer cell lines used for these studies contain BAF57.

To further identify the specific complex recruited to the MMTV promoter, we performed ChIP experiments using antibodies recognizing specific components of the BAF and PBAF complexes. Simultaneously with the recruitment of PR to the MMTV promoter after hormone treatment, we observed recruitment of Brg1, Brm, BAF250 and BAF57 (Figure 1G). In contrast, BAF180 and BAF200, two of the specific subunits of PBAF, were not recruited to the MMTV promoter after hormone treatment (Figure 1G, third and fourth rows from the bottom), while they were recruited to the RARβ2 promoter after retinoic acid treatment in U937 cells (Figure S2A). Moreover, siRNA mediated downregulation of BAF180 in T47D-MTVL cells did no influenced hormonal induction of the MMTV promoter (Figure S2B). Thus, BAF complexes containing either one of the two related ATPases together with BAF250 and BAF57 are recruited to the MMTV promoter along with PR and likely mediate promoter transactivation.

### BAF57 and BAF250 interact with progesterone receptor in T47D-MTVL cells

**Figure 2:**
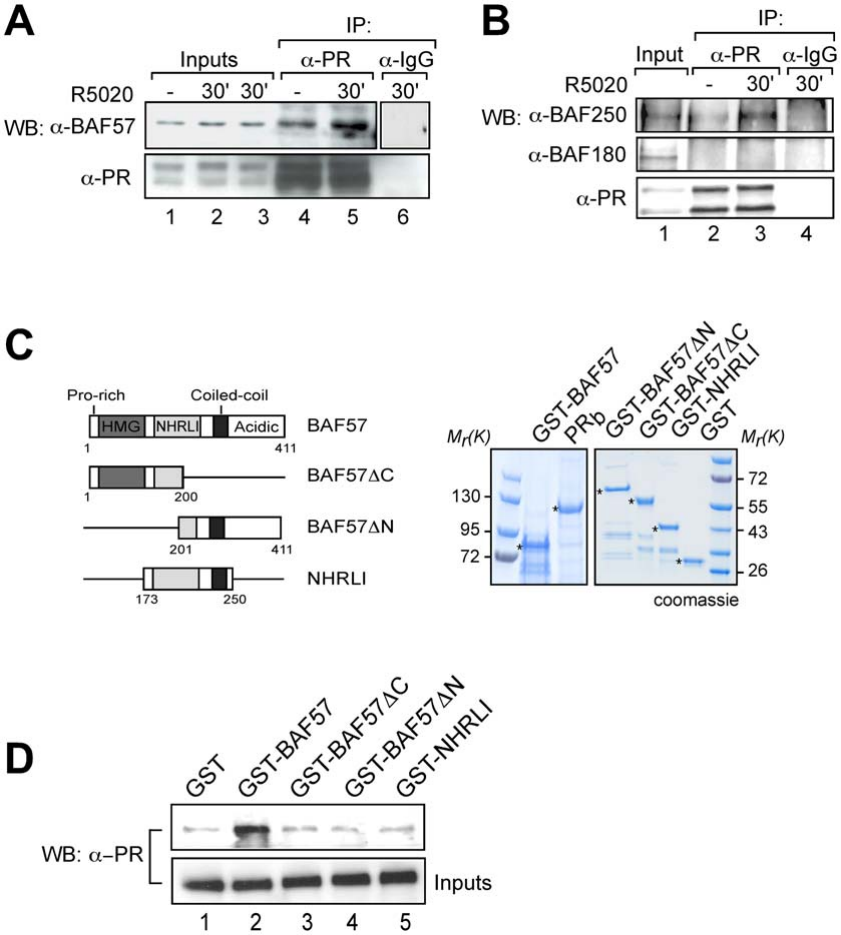
PR forms a complex with BAF in T47D-MTVL cells and binds BAF57 *in vitro*. (A) Cells were untreated (−) or treated for 30 min with R5020, lysed and immunoprecipitated either with α-PR antibody or with normal rabbit IgG as a negative control (IgG). Inputs and IPs were analyzed by western blot using α-BAF57 and α-PR, as indicated. (B) Cells were treated as in (A). Inputs and IPs were analyzed by western blot using α-BAF180, α-BAF250 and α-PR, as indicated. (C) Left: Scheme of wild type GST-BAF57 or GST-BAF57 deletion mutants used for pulldown assays. Right: Coomassie-stained gel with the level of expression of the BAF57-GST fusion proteins. (D) Recombinant PR and either wild type GST-BAF57 or GST-BAF57 deletion mutants were used for pulldown assays. Binding reactions were incubated with glutathione sepharose beads and eluted by boiling in SDS sample buffer. Input and bound material were analyzed by western blot with α-PR.

BAF57 has been shown to directly interact with the androgen and estrogen receptors [38],[39]. We used co-immunoprecipitation experiments to test whether BAF57 forms a complex with PR in cultured cells. In the absence of hormone, a certain proportion of BAF57 already coprecipitated with PR probably due to the large proportion of PR molecules already present in the nucleus in the uninduced state; however 30 minutes after hormone addition the extent of coprecipitation was increased (Figure 2A, lanes 4 and 5). In contrast, no complex of PR with the PBAF specific subunit, BAF180 was observed independently of the addition of the hormone (Figure 2B). As a positive control for this experiment we used BAF250, a known BAF specific subunit [40]. BAF250 as BAF57 also showed a hormone-dependent interaction with PR (Figure 2B, lanes 2 and 3).

The PR-BAF57 interaction is likely direct, as activated ligand-bound PR interacts with GST-BAF57 in pull-down studies (Figure 2D, lane 2). Deletion mutants lacking either the C-terminal domain or the N-terminal domain, or containing only the central NHRLI domain (Figure 2C, left panel), could not form a complex with PR in GST-pulldown experiments (Figure 2D top row, lanes 3–5).

The fact that BAF57 is present in both BAF and PBAF complexes but only BAF is recruited to the target promoters indicate that either the PR-BAF57 interaction is not sufficient for recruitment of the complex or BAF57 can only interact with PR in the context of the BAF complex but not when integrated in the PBAF complex.

### Acetylation on histone H3K14 by PCAF is required for hormonal induction

**Figure 3:**
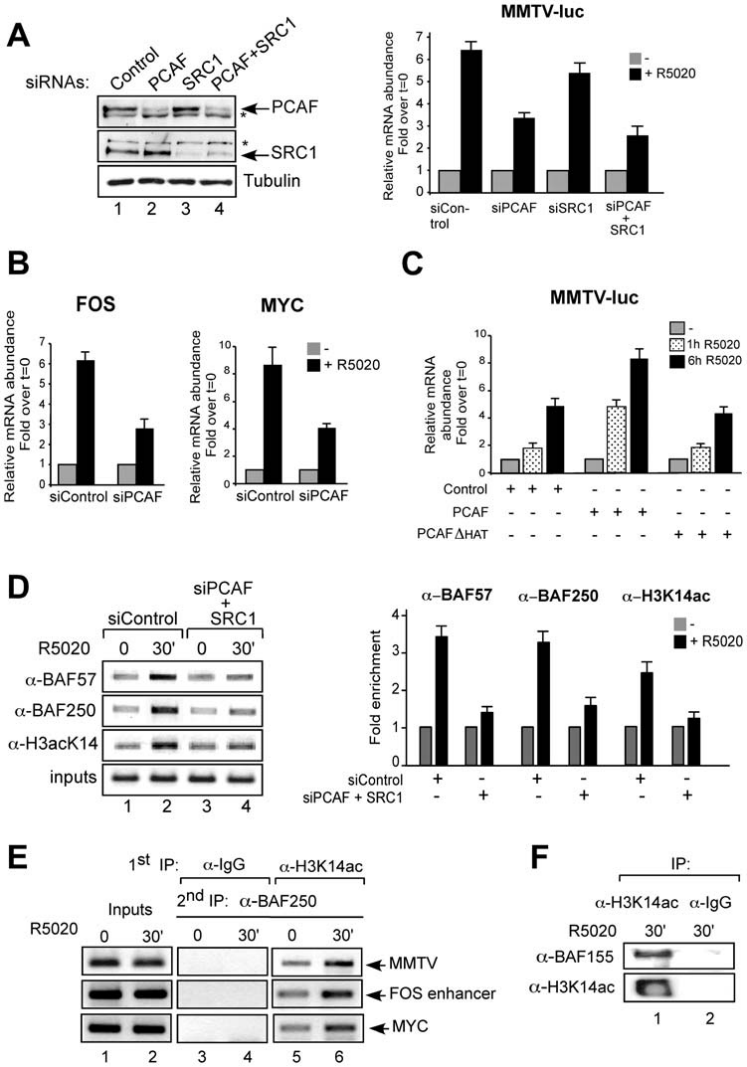
Acetylation on histone H3K14 by PCAF is essential for hormonal transactivation in T47D-MTVL cells. (A) Left: Cells were transfected with the indicated siRNAs and the levels of PCAF, SRC1, and tubulin were determined by Western blotting. The asterisks indicate inespecific bands. Right: Cells were transfected with the indicated siRNAs and treated with 10 nM R5020 for 6 h. RNA was extracted, cDNA was generated and used as template for real time PCR with luciferase oligonucleotides. The values represent the mean and standard deviation from 3 experiments performed in duplicate. (B) Cells were transfected with siRNAs, treated with 10 nM R5020 as indicated and the levels of c-Fos and c-Myc mRNAs were analyzed by RT–PCR. The values represent the mean and standard deviation from 2 experiments performed in duplicate. (C) Cells were transfected with PCAF or PCAF HAT mutant (PCAFΔHAT), treated with hormone as indicated and transcription from the MMTV promoter was determined as in (B). An empty vector was used as control. The values represent the mean and standard deviation from 2 experiments performed in duplicate. (D) Left: Cells transfected with the siRNAs and treated with hormone as indicated were subjected to ChIP assays with α-BAF57, α-BAF250 and α-K14acH3. The precipitated DNA fragments were subjected to PCR analysis to test for the presence of sequences corresponding to the MMTV nucleosome B. A representative of three independent experiments is shown. Right: quantification of the results by real time PCR from two experiments performed induplicate. (E) Cells were treated with R5020 as indicated and subjected to re-ChIP assays with α-H3K14ac and IgG (first IP) followed by α-BAF250 for the second IP. Precipiated DNA was analysed by PCR for the presence of sequences corresponding to the MMTV, c-Fos and c-Myc PR binding regions. (F) Cells were treated for 30 min with R5020, lysed and chromatin was immunoprecipitated either with α-H3K14ac or with normal rabbit IgG as a negative control (IgG). IPs were analyzed by western blot using α-BAF155 and α-H3K14ac.

As the central ATPases of the BAF complex, Brg1 and Brm, both contain bromodomains that recognize acetylated lysines [41],[42] and have been shown to bind acetylated histone H3 and H4 tails [43],[44], we wondered about possible roles of histone acetyltransferases (HATs) in hormone induction. Using siRNAs we knocked down PCAF and/or SRC1, which are known to be recruited to the MMTV promoter upon induction [6],[30] (Figure 3A). We do not have an explanation for the enhancement of the unspecific band (marked with an asterisk in Figure 3A, first row, lanes 2 and 4). When siPCAF was used, there was a 50% reduction of the MMTV induction (Figure 3A, right panel) paralleling the decrease in PCAF protein (Figure 3A, left panel), whereas siSRC1 caused only a 15% decrease (Figure 3A, right panel). The combination of the two siRNAs decreased induction by 62% (Figure 3A, right panel), indicating that PCAF is the more relevant HAT in hormonal activation of MMTV. PCAF depletion also decreased induction of other progesterone target genes such as MYC and FOS (Figure 3B), which were shown to depend on BAF.

The need for the catalytic activity of PCAF in regulating MMTV promoter was studied in T47D-MTVL cells transfected with wild type PCAF or an enzymatic deficient mutant (PCAFΔHAT). While transfection of wild type PCAF further increased the level of activity obtained after hormone addition, transfection of PCAFΔHAT did not further stimulate the MMTV promoter (Figure 3C). Although PCAF and PCAFΔHAT are expressed at similar levels (data not shown), PCAFΔHAT does not act as a dominant negative mutant possibly because its levels are not sufficient to compete with endogenous PCAF in transient transfection. Similar results were obtained when MYC and FOS genes were analysed (data not shown). Thus, the catalytic activity of PCAF is required for transcriptional activation of those progestin target genes, which induction depends on BAF. Regarding other acetyl transferases, no decrease in hormone-dependent activation of the MMTV was observed after transfection with specific siRNAs against GCN5 (data not shown).

### Acetylated H3 lysine 14 anchors the BAF complex on the promoter

We next tested the influence of histone acetylation on BAF recruitment to the MMTV promoter. In control cells hormone treatment induced increased acetylation of H3K14 accompanied by recruitment of BAF57 and BAF250, whereas knockdown of PCAF and SRC1 abrogated K14 acetylation and markedly reduced BAF57 and BAF250 loading on the promoter (Figure 3D, compare lanes 1–2 vs 3–4; quantification by real time PCR is shown in the right panel). To test whether H3K14 acetylation and BAF binding take place on the same promoter, we performed sequential ChIP (re-ChIP) experiments in the MMTV, FOS and the MYC promoters. Hormone treatment induced increased acetylation of H3K14 accompanied by recruitment of BAF250 to the corresponding promoters (Figure 3E, lanes 5 and 6), indicating that the H3K14 acetylation and BAF recruitment occur in the same genomic region. In contrast, control re-ChIPs performed with irrelevant IgG as first antibody showed no amplification product (Figure 3E, lanes 3 and 4).

To test whether the association of H3K14 acetylation and BAF is a general phenomenon, we performed co-immunoprecipitation of total hormone-treated chromatin followed by western blot. This seems to be the case, as immunoprecipitation with antibody against acetylated H3K14 co-precipitated BAF155 (Figure 3F) and Brg1 (data not shown).

**Figure 4:**
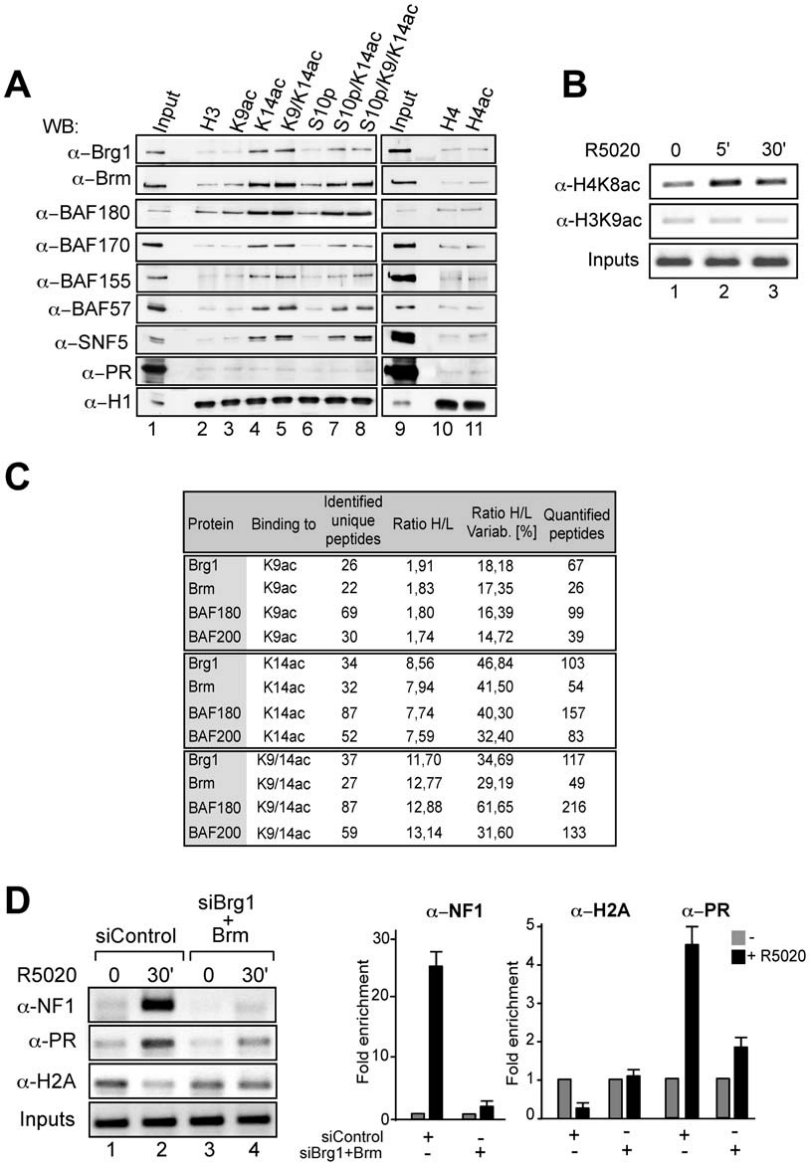
The BAF complex binds preferentially to histone H3K14ac and promotes H2A displacement. (A) Nuclear extracts derived from T47D-MTVL cells were used for pulldown experiments with the indicated H3 and H4 tail peptides coupled to beads. Immunoblotting was performed for the presence of components of the BAF and PBAF complex, histone H1 and PR. (B) Cells were untreated (0) or treated for 5 and 30 min with 10 nM R-5020 and subjected to ChIP assays with α-H4K8ac and α-H3K9ac specific antibodies. The precipitated DNA fragments were subjected to PCR analysis to test for the presence of sequences corresponding to the MMTV nucleosome B. Input material (1%) is shown for comparison. A representative of three independent experiments is shown. (C) Quantification of the interaction of selected human SWI/SNF subunits with the indicated H3 tail acetylated peptides. SILAC ratios represent the relative abundance of the ‘heavy’ (modified) to the ‘light’ (unmodified) peptide. The variation (in %) and the number of quantified peptides are indicated. (D) Left: T47D-MTVL cells were transfected with siRNAs and treated with hormone as indicated, subjected to ChIP assays using α-NF1, α-PR and α-H2A and primers for MMTV nucleosome B. Right: quantification of the results by real time PCR. A representative of three independent experiments is shown.

We next used pull-down experiments with T47D-MTVL nuclear extracts and biotinylated peptides of H3 and H4 tails containing various modifications to test whether H3K14 acetylation influences the binding affinity of the BAF complex for histone tails. Histone H1 was used as loading control (Figure 4A, lower row). We detected binding to histone tails containing H3K14ac of nearly all subunits of the BAF and PBAF complexes, namely Brg1, Brm, BAF170, BAF155, BAF57, BAF180 and SNF5 (Figure 4A, lanes 4, 5, 7, and 8). Similar results were also obtained using HeLa nuclear extracts (data not shown). Moreover, binding of BAF and PBAF complexes to H3 peptides depends solely on H3K14ac and it is not affected by acetylation, phosphorylation or methylation of adjacent residues. Peptides carrying additional modifications, such as K4me3, K9ac, K9me3, or S10p, exhibited similar affinity for BAF subunits as those carrying only K14ac (Figure 4A lanes 5, 7, 8 and data not shown). H3 peptides acetylated at K9 and pan-acetylated (K5, 8, 12, 16) H4 peptides did not exhibit preferential BAF affinity (Figure 4A lanes 3, 10 and 11). Although H4K8 acetylation takes place very early after progesterone addition to T47D-MTVL cells (Figure 4B, upper panel), we found here no binding of the BAF complex to acetylated H4 peptides. The PBAF specific subunit, BAF180 showed the same behaviour as the BAF subunits when incubated with the modified peptides (Figure 4A, third row from the top). It is important to note, that we detected a faint and consistent binding of PR to all tested H3 and H4 peptides without any preference for particular epigenetic modification, indicating that PR is not acting as a bridging factor for the binding of BAF complex to the K14 acetylated peptides (Figure 4A, second row from the bottom). A similar behaviour was observed for PCAF without any preference for the tested modifications (data not shown).

Next, we quantify the interaction of the BAF subunits to H3 acetylated peptides by *s*table *i*sotope *l*abeling by *a*mino acids in *c*ell culture (SILAC) [45]. Nuclear extracts derived from human HeLaS3 cells grown in light or heavy medium were incubated with immobilized histone peptides in the nonacetylated (H3) and acetylated form, respectively (Figure S3A). We identified BAF180, BAF200, Brg1 and Brm as selective interactors for H3K9ac, H3K14ac and H3K9acK14ac. The SILAC ratio (Heavy/Light, that is modified/unmodified) for Brm was 1.8 for H3K9ac and 7.9 for H3K14ac (Figure 4C and Figure S3), indicating that BAF complex is attached preferentially to K14 acetylated peptides and confirming the results obtained with peptide pull downs (Figure 4A). Higher SILAC ratios with H3K14ac peptide were also obtained for Brg1 and for the PBAF specific subunits, BAF180 and BAF200 (Figure 4C). An additive effect on binding was observed when the double modification H3K9acK14ac was used (Figure 4C). However, we did not find an increase in H3K9ac on the MMTV promoter following hormone induction (Figure 4B, lower panel). These results highlight the role of K14 acetylation as the main modification responsible for BAF and PBAF binding.

Knocking down Brg1 and Brm reduces the interaction of BAF57 and BAF155 with H3K14ac peptides without affecting the intracellular levels of their complex with BAF170 (Figure S4 and data not shown), indicating that the bromodomains of the ATPases contribute to the anchoring of the BAF complex at H314Kac containing sites.

### The BAF complex facilitates NF1 binding by mediating histone H2A/H2B displacement

Next we used knockdown experiments to test whether BAF is involved in NF1 binding to the MMTV promoter, a critical step in hormonal induction [35]. The NF1 family of transcription factors in vertebrates is composed of four different genes: NF1A, NF1B, NF1C and NF1X [46], of which NF1C most abundantly expressed in mammary gland and is involved in reciprocal and sequential synergism with hormone receptors [47],[48]. In control cells, NF1C bound to the MMTV promoter in response to hormone, while binding was diminished upon Brg1 and Brm knockdown (Figure 4D, first row, compare lanes 1–2 vs 3–4; quantification by real time PCR is shown in the right panel), suggesting that BAF complexes are necessary for NF-1 to access the promoter in response to hormone.

To investigate how BAF action facilitates NF1 binding we investigated its involvement in the localized displacement of histones H2A and H2B dimers, which is observed after hormone induction [6]. In cells transfected with control siRNA, recruitment of PR 30 minutes after hormone addition (Figure 4D, second row, lanes 1–2) is accompanied by displacement of histone H2A (Figure 4D, third row, lanes 1–2). Depletion of Brm and Brg1 diminished PR binding and abolished H2A displacement (Figure 4D, lanes 3–4; quantification by real time PCR is shown in the right panel). Thus, BAF complexes are required for PR binding and H2A/H2B histone displacement, a requisite for NF1 binding. It is likely that the residual PR bound in the absence of BAF corresponds to receptor interacting with the exposed HRE1 [27].

## Discussion

The results of this study contribute to a better understanding of the molecular mechanisms of promoter activation by progesterone. We have shown previously that PR interacts with an exposed HRE on the surface of a nucleosome on the MMTV promoter [27] and recruits Brg1/Brm-containing complexes [6]. Here we demonstrate the need of the hSWI/SNFα complex, known as BAF, for activation of MMTV and other progesterone target genes. We show that BAF is recruited at least in part *via* an interaction between PR and the BAF57 subunit of the complex. In a previous report we have described that acetylation of H3K14 in response to progestins [30]. Now we show that H3K14ac, likely generated by PCAF, anchors the BAF complex, suggesting a mechanism for the cooperation between two types of chromatin remodeling activities. The recruited BAF catalyzes the ATP dependent displacement of histones H2A/H2B needed for NF1 to gain access to the promoter site. Thus synergism between the two transcription factors PR and NF1 is mediated by a cooperation between two chromatin remodeling machines, BAF and PCAF.

### BAF interacts with PR and is necessary for induction of progesterone target genes in breast cancer cells

Activation of several hormone sensitive promoters exhibits SWI/SNF-dependence reflecting the requirement of chromatin remodeling [40], [49]–[51]. There is evidence that glucocorticoid receptor recruits a Brg1-containing complex to promoters via protein-protein interaction with BAF250 [40], whereas androgen receptor (AR) and ERα can directly bind BAF57 [39],[51]. We have described a progesterone dependent recruitment of Brg1 and Brm to the MMTV promoter in breast cancer cells and have shown that yeast SWI/SNF can displace H2A/H2B dimers from MMTV recombinant nucleosomes [6], but the nature of the complex recruited in intact cells and its function in gene activation was not known.

The human SWI/SNF complexes can have either Brm or Brg1 as ATPase subunits [52]. Although the two ATPases exhibit certain differences in their biological activities [53],[54], they can partly compensate for each other in mouse cells [55]. We found that in T47D-MTVL cells depletion of Brm increases Brg1 levels and viceversa. This could be due to the existence of an excess of both free ATPases in equilibrium with the complex bound forms. If one of the ATPase is depleted the other will be incorporated to a higher extent in the SWI/SNF complex thus becoming more resistant to proteolytic degradation. A similar finding has been reported in mice lacking Brm [56].

The BAF complex is recruited to the MMTV promoter within minutes after progestin treatment, likely through a direct interaction with the activated PR. The fact that PR was unable to form a complex with PBAF indicates that the receptor can discriminate between these two related machineries and promotes BAF recruitment to progestin target promoters. Although BAF57 is present in BAF and PBAF complexes it is possible that hormone-dependent interaction with PR is only possible in the context of the BAF complex. Similar results have been previously reported for AR and ERα [38],[39],[51]. Depletion of BAF250 has a similar effect on MMTV induction as depletion of both Brg1 and Brm, confirming the importance of the BAF complex. BAF dependence is observed with other progesterone responsive genes, such as FOS and MYC, indicating that chromatin remodeling by BAF plays a more general role in progesterone gene regulation. However, as exemplified by the cyclin D1 gene, not all hormone responsive genes required BAF for induction, indicating the existence of alternative pathways for hormonal gene regulation. It is likely that other ATP-dependent remodeling complexes participate in regulation of MMTV and other progesterone regulated genes. In minichromosomes reconstituted in *Drosophila* embryo extracts NURF mediates synergism between recombinant PR and NF1 in a SWI/SNF independent manner [35]. The difference with our present results may reflect the different nature of chromatin in breast cancer cells and Drosophila embryo, such as lack of linker histones and transcriptional activity. We know that hSnf2H is recruited to the MMTV promoter 30 min after progesterone treatment of T47D-MTVL cells but its role in hormonal induction remains to be established [30].

### Acetylation on histone H3K14 by PCAF is essential for hormonal induction by anchoring BAF to the promoter

The role of histone acetylation on MMTV activity has been controversial. We have observed that low concentration of HDAC inhibitors leading to moderate increase in acetylation, enhance MMTV transcription [37], whereas high concentrations completely abolish transcription [37],[57]. In previous reports, we have demonstrated the co-recruitment of PCAF and SRC1 to the MMTV promoter following progestin treatment [6],[30]. The siRNA knockdown results presented here demonstrate that PCAF is one of the main HATs acting on the MMTV promoter, that its levels correlated with the extent of H3K14 acetylation, and that acetylation is necessary and important for progesterone activation of the MMTV promoter.

There have also been controversies over the nature of the histone modifications influencing binding of the SWI/SNF complex [58]. It was first claimed that acetylation of H4K8 recruits the SWI/SNF complex via Brg1, whereas acetylation of H3K9 and H3K14 is only important for the recruitment of TFIID general transcription factors [43]. Two independent recent reports demonstrated that the bromodomain-containing ATPase Brg1 has the highest binding affinity to acetylated H3K14 peptide or doubly acetylated H3K9/K14 peptide, whereas binding to acetylated H4K8 peptide is insignificant [59],[60]. Although H4 acetylation takes place very early after progesterone addition to T47D-MTVL cells (Figure 4B), we found here no binding of the BAF complex to acetylated H4 peptides (Figure 4A). In contrast, we show that components of the BAF complex bind to peptides containing acetylated H3K14, alone or in combination with other histone modifications. Furthermore, we quantify the interaction of components of BAF and PBAF to H3 acetylated peptides by SILAC. Our results indicate that compared to H3K9ac, H3K14ac is the major contributor to anchoring of these chromatin-remodeling complexes to nucleosomes. Several subunits of BAF and PBAF contain bromodomains that can bind acetylated residues. Obvious candidates are Brg1 and Brm, but other subunits, such as BAF180, could cooperate on the recognition of H3K14ac by PBAF. Despite the fact that both BAF and PBAF can bind acetylated K14, only BAF is recruited to the MMTV promoter, indicating that H3K14ac participates in anchoring or retention of the recruited complex but is not sufficient for recruitment.

The colocalization of BAF and H3K14ac (Figure 3F) could reflect a general role of this histone mark to BAF anchoring in chromatin, but other explanations are possible. For instance, a common transcription factor could recruit BAF and HATs to the same chromatin regions. Alternatively, components of the BAF complex could be important for HATs recruitment. A combination of these hypotheses is also possible.

### BAF mediates nucleosome remodeling leading to H2A/H2B displacement that facilitates NF1 binding

Following hormone treatment, PR is phosphorylated, forms a complex with activated Erk and Msk1 and this ternary complex binds the exposed HRE1 on the surface of the MMTV promoter nucleosome leading to modification of the H3 tail and displacement of a repressive complex [30]. PCAF is also recruited to the promoter and acetylation of H3K14 is observed 5 min after hormone treatment [30]. The activated ternary complex pPR-pErk-pMsk1 recruits BAF that initiates nucleosome remodeling, since inhibiting Erk or Msk1 activation blocks BAF recruitment and the subsequent steps. We have not addressed the initial kinetics of acetylation and BAF recruitment in sufficient detail and cannot propose a precise order of events. Moreover, we cannot exclude that PCAF interacts with BAF and that both complexes are recruited together.

**Figure 5:**
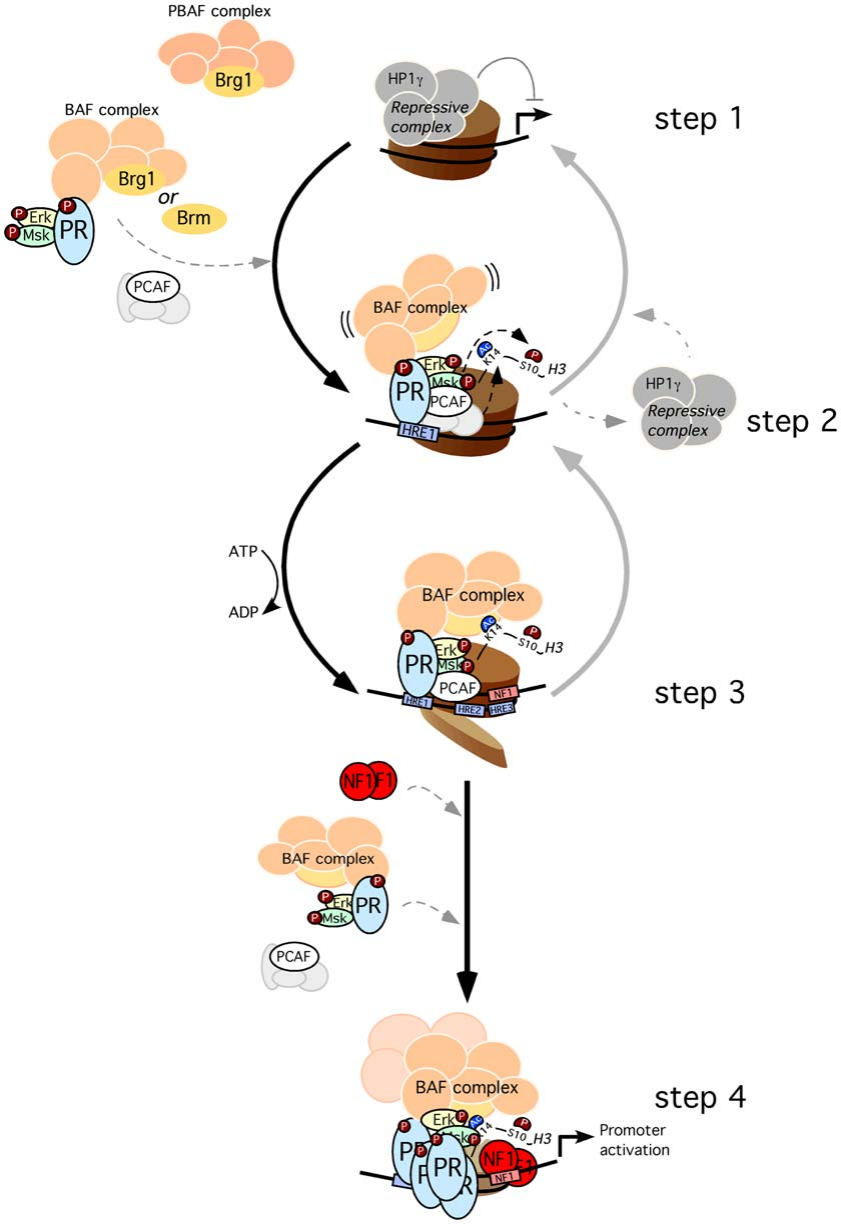
Model for the initial steps of MMTV promoter activation. Before hormone addition the MMTV promoter is silent and associated with a repressive complex that includes HP1γ (step 1). After hormone addition the activated complex of pPR-pErk-pMsk, as well as PCAF and BAF, are recruited to the MMTV promoter. For simplicity PR is shown as a monomer, though the active form is a homodimer. Msk and PCAF phosphoacetylates H3 leading to H3S10phK14ac (step 2). This modification displaces the repressive complex and anchors the BAF complex, enabling ATP-dependent H2A/H2B displacement (step 3). The nucleosome opening facilitates NF1 binding generating a stable platform that exposes previously hidden HREs for the recruitment of additional PR and BAF complexes, coactivator and eventually promoter activation (step 4). Depletion of BAF prevents progression of the activation process to step 2: no histone displacement is observed, NF1 cannot bind and consequently less PR/BAF complexes are bound to the promoter. Depletion of PCAF has a similar effect, most likely by labilization of BAF binding, blocking the activation process at step 2.

In T47D-MTVL cells the BAF mediated chromatin remodeling event is a localized displacement of dimers of H2A/H2B from promoter nucleosome B, a catalytic activity exhibited by the purified yeast SWI/SNF complex on recombinant MMTV nucleosomes [6]. An earlier study with recombinant nucleosomes and SWI/SNF complexes from yeast suggest that both BAF and PBAF complexes would have the capacity to displace histones H2A/H2B as they share the relevant subunit, Swi3p [36]. This remodeling event is a prerequisite for enabling access of NF1 to its binding site in the MMTV promoter following hormone treatment, as demonstrated by the lack of H2A displacement and NF1 binding in BAF-depleted cells. We have previously shown that NF1 binding stabilizes the open conformation of the nucleosome allowing binding of further PR molecules to the internal HREs and subsequent activation of transcription [35]. A model representing our present view of the initial steps in progesterone activation of the MMTV promoter is shown in Figure 5. The model underlines the feed-forward cyclical nature of the activation process and explains why interfering with any of the initial steps has consequences for binding of all factors involved, PR, NF1, BAF and PCAF.

Briefly, after hormone induction activated PR binds first to the exposed HRE1 in a process that does not require chromatin remodeling, as this site is accessible [28]. Along with the activated PR, kinases, BAF and PCAF are recruited to the promoter chromatin. Histone H3 S10 phosphorylation and K14 acetylation promote displacement of an HPIγ-containing repressive complex [30] and BAF anchoring, respectively. Now, BAF can displace histones H2A/B and thus facilitate NF1 binding. Bound NF1 stabilize the open conformation of the remodeled nucleosome exposes the previously hidden HREs allowing binding of further PR-BAF-kinases complexes and PCAF. Binding of PR to these previously hidden HREs is critical for the MMTV activation.

Finally, it is worth noting that we have not mentioned in these studies the possible role of linker histones. Our previous results [61] and those of other groups [62]–[64], suggest that changes histone H1 stoichiometry and phosphorylation by CyclinA/Cdk2 take place at different time points during the hormonal induction and are important for transcriptional activation. Future studies will be required to clarify the relationship of these changes to those reported in this study.

## Materials and Methods

### Cell culture and hormone treatments

T47D-MTVL breast cancer cells carrying one stably integrated copy of the luciferase reporter gene driven by the MMTV promoter [27] were routinely grown in RPMI 1640 medium supplemented with 10% FBS, 2 mM L-glutamine, 100 U/ml penicillin and 100 µg/ml streptomycin. For the experiments, cells were plated in RPMI medium without phenol red supplemented with 10% dextran-coated charcoal treated FBS (DCC/FBS) and 48 h later medium was replaced by fresh medium without serum. After 24 h in serum-free conditions, cells were incubated with R5020 (10 nM) or vehicle (ethanol) for different times at 37°C.

### Transient transfections

Cells were cultured into 6-well plates at a density of 4×10^5^ cells/well and treated as indicated above. Transient transfections were performed using Lipofectamine 2000 (Invitrogen). cDNAs expressing PCAF and its deacetylase defective mutant (PCAF ΔHAT) were kindly provided by Tony Kouzarides (Cambridge, UK).

### Chromatin Immunoprecipitation (ChIP) and re-ChIP assays in cultured cells

ChIP assays were performed as described [65] using the NF1 specific antibody (gift from Dr Naoko Tanese), the H2A antibody (gift from Stefan Dimitrov), anti-BAF180 (gift from Dr Weidong Wang), anti-PR (H190) and anti-Brg1 (H88), both from Santa Cruz, anti-SMARCA2/BRM (ab15597) from Abcam, anti-BAF57/SMARCE1 from Bethyl and anti-acetyl(Lys14)-Histone H3 and anti-BAF250 from Upstate. Quantification of chromatin immunoprecipitation was performed by real time PCR using Roche Lightcycler (Roche). The fold enrichment of target sequence in the immunoprecipitated (IP) compared to input (Ref) fractions was calculated using the comparative Ct (the number of cycles required to reach a threshold concentration) method with the equation 2^Ct(IP)−Ct(Ref)^. Each of these values were corrected by the human b-globin gene and referred as relative abundance over time zero. Primers sequences are available on request. For re-ChIP assays, immunoprecipitations were sequentially washed as previously described [66]. Complexes were eluted with 10 mM DTT at 37°C for 30 min, diluted 50 times with dilution buffer, and immunoprecipitated with the indicated antibodies. The antibodies used for ChIPs assays are listed in Figure S5.

### RNA interference experiments

All siRNAs were transfected into the T47D-MTVL cells using Lipofectamine 2000 (Invitrogen). After 48 h the medium was replaced by fresh medium without serum. After one day in serum-free conditions, cells were incubated with R5020 (10 nM) or vehicle (ethanol) for different times at 37°C. The down-regulation of Brg1, Brm, NF1, BAF57, BAF250, PCAF and SRC1 expression was determined by Western blotting. The siRNAs used are listed in Figure S5.

### RNA extraction and RT–PCR

Total RNA was prepared and cDNA generated as previously described [30]. Quantification of LUC and GAPDH gene products was performed by real time PCR. Each value calculated using the standard curve method was corrected by the human GAPDH and expressed as relative RNA abundance over time zero. Primer sequences are available on request.

### Coimmunoprecipitation assay

Cells were lysed and cell extracts (2 mg) were incubated with protein G/A agarose beads previously coupled with 6 µg of the corresponding antibodies or an unspecific control antibody. The immunoprecipitated proteins (IP) were eluted by boiling in SDS sample buffer. Inputs and IPs were analyzed by western blot using BRG1, BRM, BAF155, BAF170 and H3K14ac specific antibodies.

### Peptide pull down assays

Nuclear extracts from T47DMTVL breast cancer cells were prepared as described [67]. Peptide pull down assays were performed as described previously [68], with the exception of using 100 µg of nuclear extract during incubation of peptide-bound beads. Synthetic biotinylated H3 and H4 peptides were either purchased from Upstate or were kind gifts from M. Vermeulen. For Western immunoblotting, antibodies against BRG1, BAF170, BAF155, PR (Santa Cruz), BRM (Abcam), BAF57, SNF5 (Bethyl) and H1 (Upstate 05-457) were used.

### Mass spectrometry of proteins

After trypsin digestion of gel slices peptides were extracted, desalted using stage tips [69] and analyzed using a nano-HPLC Agilent 1100 nanoflow system connected online to an LTQ-Orbitrap mass spectrometer (Thermo Fisher, Bremen). The mass spectrometer was operated in the data-dependent mode to automatically switch between MS and MS^2^. The instrument was operated with ‘lock mass option’ as recently described [70]. Survey spectra were acquired with 60,000 resolution in the orbitrap, while acquiring up to five tandem mass spectra in the LTQ part of the instrument. The raw data files were analyzed with an in-house developed quantitative proteomics software MaxQuant, version 1.0.12.5 [71], in combination with the Mascot search engine (Matrix Science). The data was searched against a decoy human IPI database 3.37 including common contaminants. False discovery rates, both at the peptide and protein level were set to 1%. Minimal peptide length was set to 6 amino acids. False positive rates for peptides are calculated as described in [72].

## Supporting Information

Figure S1 Differential expression of BAF proteins in T47D-MTVL breast cancer cells. Nuclear extracts from T47D-MTVL cells were analyzed by western blotting with antibodies specific for the individual BAF proteins.
(0.39 MB TIF)

Figure S2 Knock down of BAF180 does not affect MMTV transcriptional activity. (A) BAF180 is recruited to the RARβ2 promoter. U937 cells (human promyelocitic leukemia cell line) were untreated (−) or treated (+) for 4 hs with retinoic acid (RA) and subjected to ChIP assays with BAF200 and BAF180 specific antibodies. The precipitated DNA fragments were subjected to PCR analysis to test for the presence of sequences from −665 to −308 corresponding to the RARβ2 promoter. Lower Panel: expression of BAF180 and BAF200 proteins in U937 cells. (B) Knock down of BAF180 does not affect MMTV transcriptional activity. Left: T47D-MTVL cells were transfected either with control siRNA or with siRNA against BAF180. After 48 h the medium was replaced by fresh medium without serum. After one day in serum-free conditions, cells were lysed and the levels of BAF 180 and tubulin were determined by Western blotting. Right: T47D-MTVL cells were transfected with Control or BAF 180 siRNAs in RPMI medium and cultured for 48 h. After one day in serum-free conditions, cells were incubated with 10 nM R5020 for 2 hs and total RNA was prepared, cDNA was generated and used as template for real time PCR with specific Luciferase and GAPDH primers. Each luciferase mRNA value was corrected by the GAPDH mRNA level and is expressed as relative RNA abundance over time zero. The values represent the mean and standard deviation from 3 experiments performed in duplicate.
(0.17 MB TIF)

Figure S3 Schematic representation of the SILAC-based histone pull down approach. (A) SILAC peptide pull-down using unmodified H3 (grey ovals), H3K9ac (B, blue ovals), and H3K14ac (C, red ovals) peptide. The spectra show the relative binding of Brm (1), BAF200 (2), Polybromo/BAF180 (3), and Brg1 (4) to unmodified and modified peptides.
(1.17 MB TIF)

Figure S4 Brg1 and Brm are required for the interaction of BAF and PBAF complexes with the H3K14ac mark. (A) Nuclear extracts derived from T47D-MTVL cells transfected with control and Brg1 and Brm siRNAs were used for pulldown experiments with the indicated H3 tail peptides coupled to beads. Immunoblotting was performed for the presence of BAF57 and H1. (B) Nuclear extracts derived from T47D-MTVL cells transfected with control and Brg1 and Brm siRNAs were used for immunoprecipitation either with BAF57 antibody or with normal rabbit IgG as a negative control (IgG). The immunoprecipitates (IP) were analyzed by western blotting with BAF155 and BAF170 specific antibodies.
(0.22 MB TIF)

Figure S5 siRNAs and antibodies used for ChIP assays in the present study.
(0.67 MB TIF)
