# Supplementary figures and images for: Two Chromatin Remodeling Activities Cooperate during Activation of Hormone Responsive Promoters
Source: PLoS Genet. 2009 Jul 17;5(7):e1000567. doi: 10.1371/journal.pgen.1000567 (PMC2704372; doi:10.1371/journal.pgen.1000567)

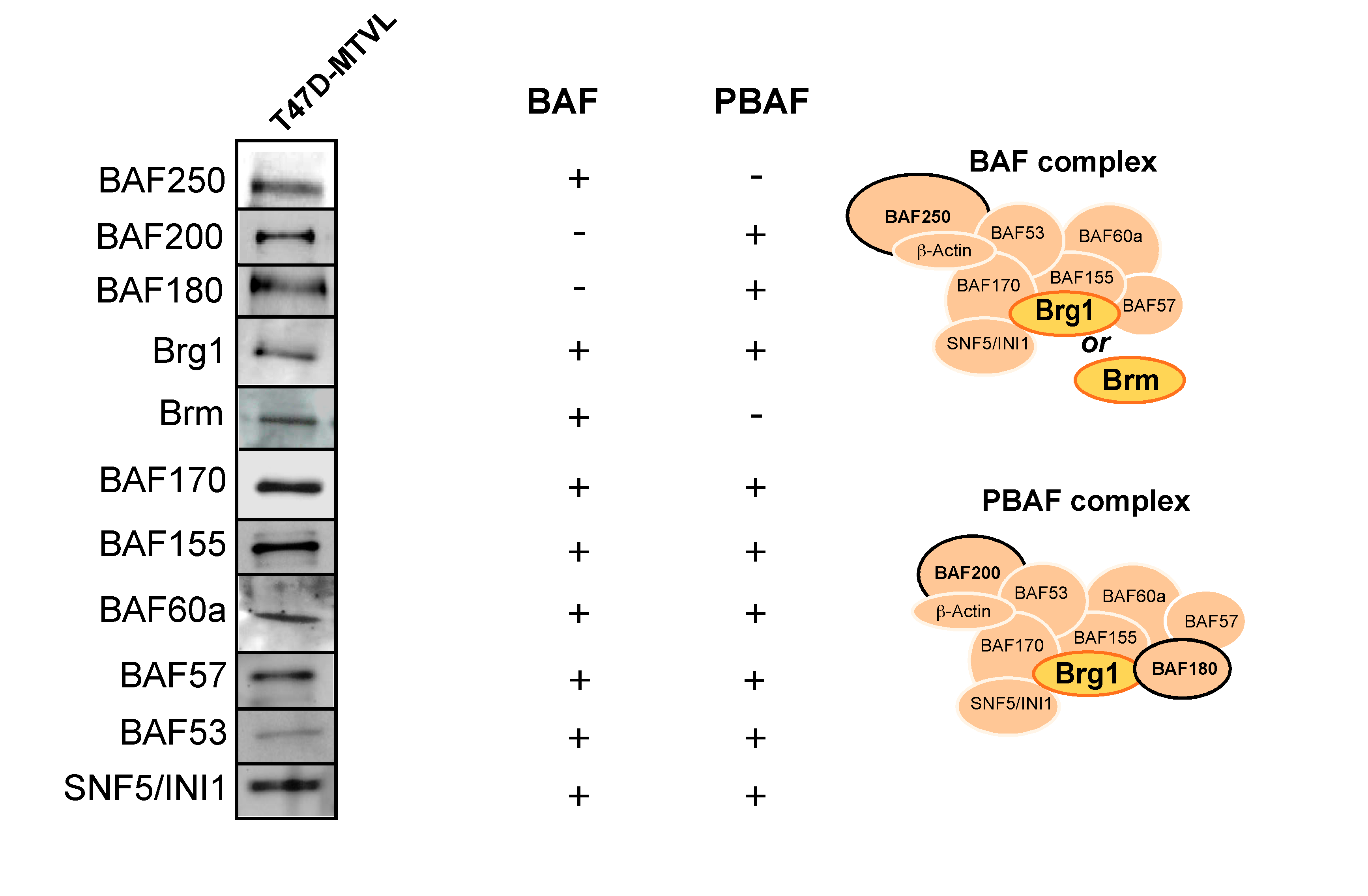

Supplement: Figure S1 — Differential expression of BAF proteins in T47D-MTVL breast cancer cells. Nuclear extracts from T47D-MTVL cells were analyzed by western blotting with antibodies specific for the individual BAF proteins. (0.39 MB TIF) [file pgen.1000567.s001.tif]

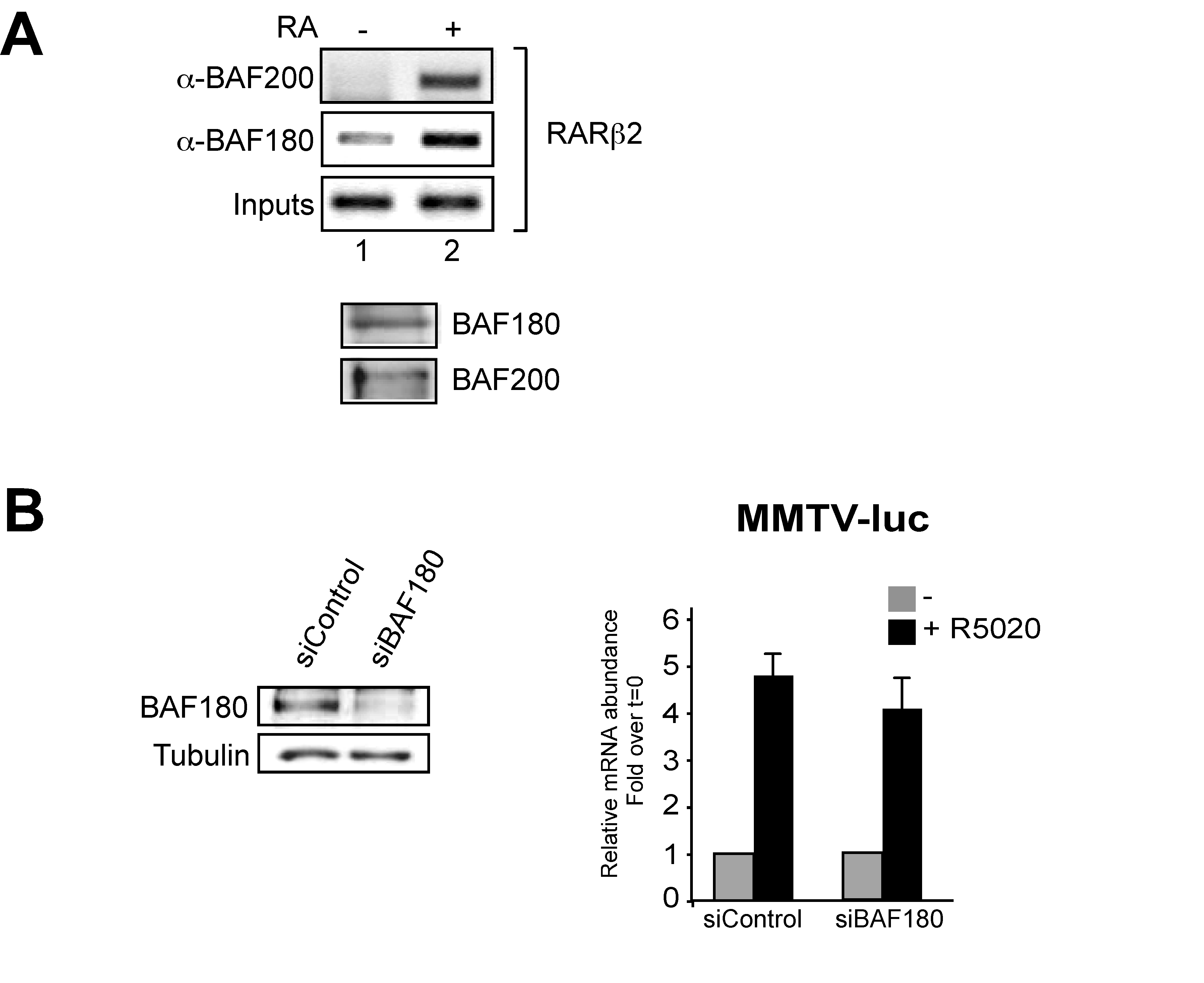

Supplement: Figure S2 — Knock down of BAF180 does not affect MMTV transcriptional activity. (A) BAF180 is recruited to the RARβ2 promoter. U937 cells (human promyelocitic leukemia cell line) were untreated (−) or treated (+) for 4 hs with retinoic acid (RA) and subjected to ChIP assays with BAF200 and BAF180 specific antibodies. The precipitated DNA fragments were subjected to PCR analysis to test for the presence of sequences from −665 to −308 corresponding to the RARβ2 promoter. Lower Panel: expression of BAF180 and BAF200 proteins in U937 cells. (B) Knock down of BAF180 does not affect MMTV transcriptional activity. Left: T47D-MTVL cells were transfected either with control siRNA or with siRNA against BAF180. After 48 h the medium was replaced by fresh medium without serum. After one day in serum-free conditions, cells were lysed and the levels of BAF 180 and tubulin were determined by Western blotting. Right: T47D-MTVL cells were transfected with Control or BAF 180 siRNAs in RPMI medium and cultured for 48 h. After one day in serum-free conditions, cells were incubated with 10 nM R5020 for 2 hs and total RNA was prepared, cDNA was generated and used as template for real time PCR with specific Luciferase and GAPDH primers. Each luciferase mRNA value was corrected by the GAPDH mRNA level and is expressed as relative RNA abundance over time zero. The values represent the mean and standard deviation from 3 experiments performed in duplicate. (0.17 MB TIF) [file pgen.1000567.s002.tif]

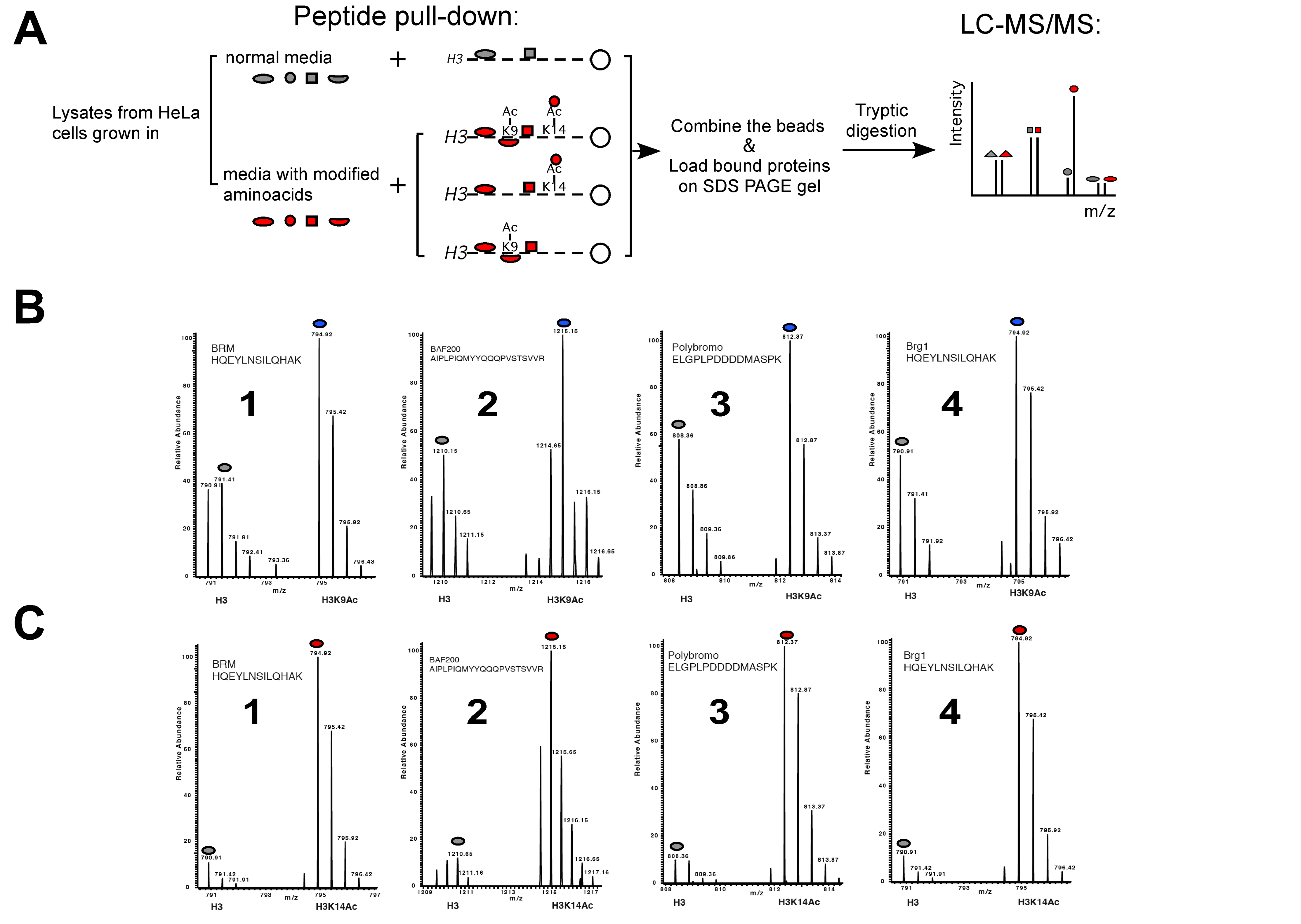

Supplement: Figure S3 — Schematic representation of the SILAC-based histone pull down approach. (A) SILAC peptide pull-down using unmodified H3 (grey ovals), H3K9ac (B, blue ovals), and H3K14ac (C, red ovals) peptide. The spectra show the relative binding of Brm (1), BAF200 (2), Polybromo/BAF180 (3), and Brg1 (4) to unmodified and modified peptides. (1.17 MB TIF) [file pgen.1000567.s003.tif]

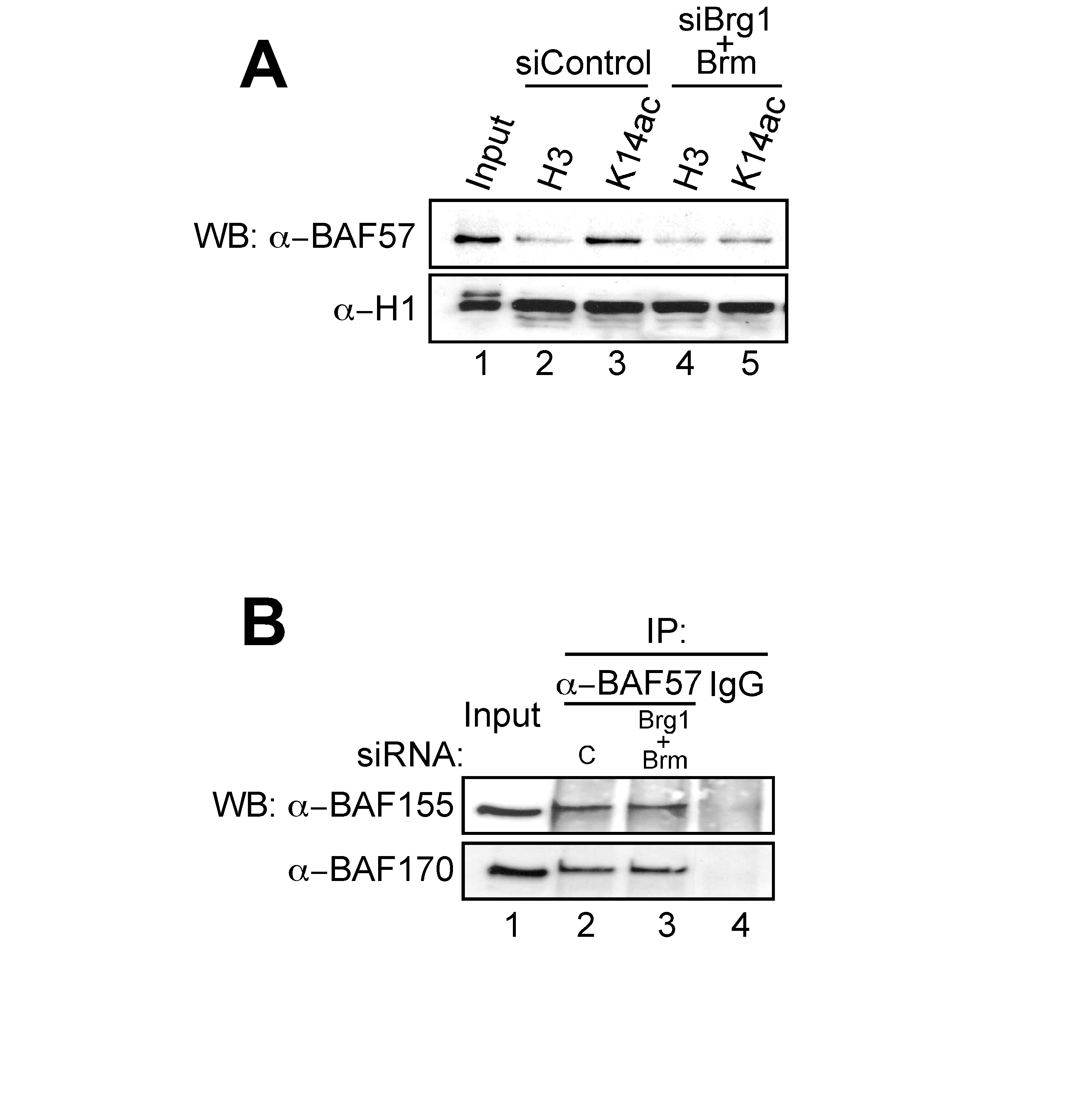

Supplement: Figure S4 — Brg1 and Brm are required for the interaction of BAF and PBAF complexes with the H3K14ac mark. (A) Nuclear extracts derived from T47D-MTVL cells transfected with control and Brg1 and Brm siRNAs were used for pulldown experiments with the indicated H3 tail peptides coupled to beads. Immunoblotting was performed for the presence of BAF57 and H1. (B) Nuclear extracts derived from T47D-MTVL cells transfected with control and Brg1 and Brm siRNAs were used for immunoprecipitation either with BAF57 antibody or with normal rabbit IgG as a negative control (IgG). The immunoprecipitates (IP) were analyzed by western blotting with BAF155 and BAF170 specific antibodies. (0.22 MB TIF) [file pgen.1000567.s004.tif]

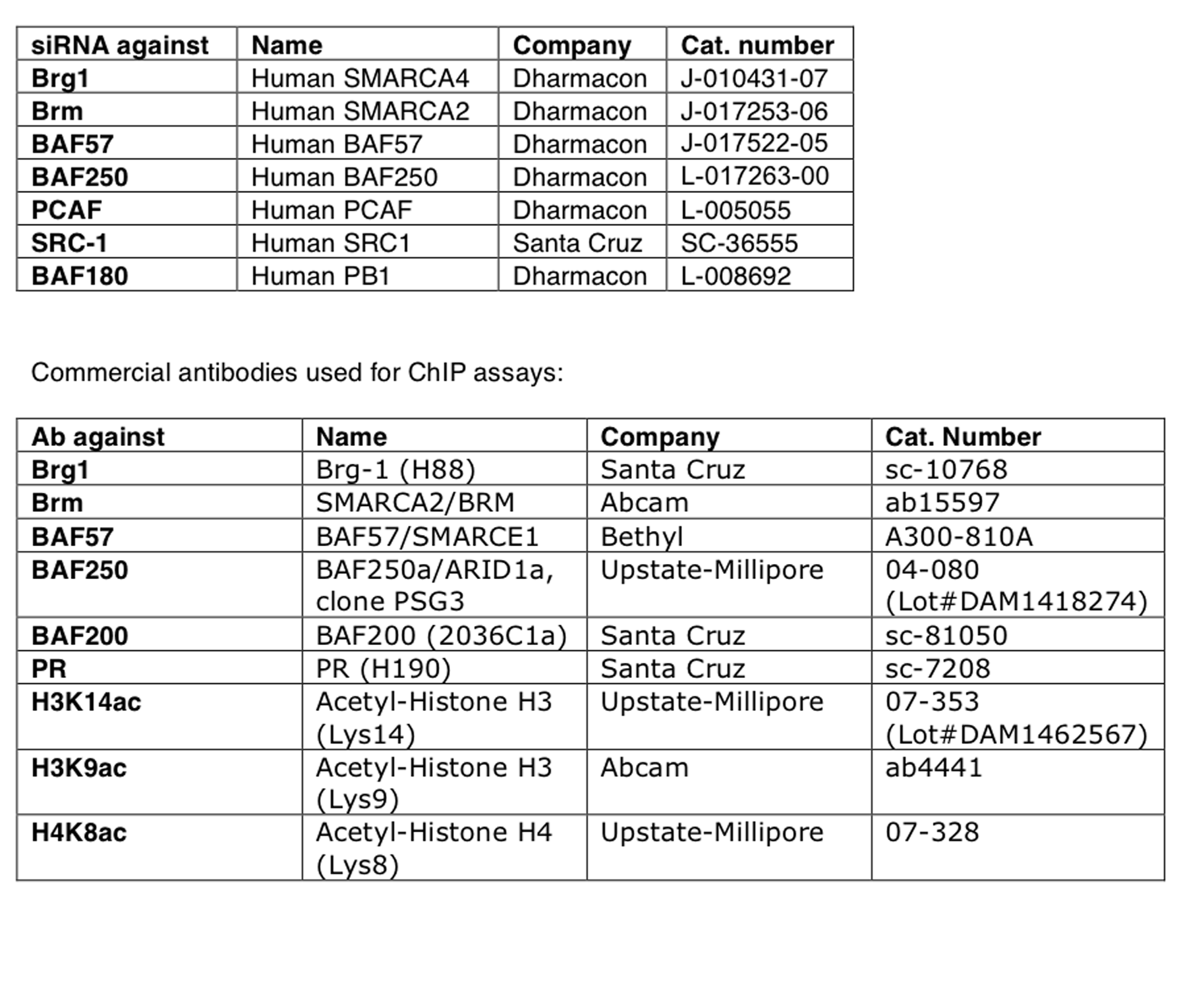

Supplement: Figure S5 — siRNAs and antibodies used for ChIP assays in the present study. (0.67 MB TIF) [file pgen.1000567.s005.tif]
